# Cardiac workload and skeletal muscle oxygenation during incremental exercise in healthy subjects

**DOI:** 10.14814/phy2.70456

**Published:** 2025-07-07

**Authors:** Tommi Jeskanen, Rasmus I. P. Valtonen, Venla P. Ylinen, Jan Nissinen, Mikko P. Tulppo

**Affiliations:** ^1^ Research Unit of Biomedicine and Internal Medicine, Faculty of Medicine University of Oulu Oulu Finland; ^2^ Medical Research Center Oulu Oulu University Hospital and University of Oulu Oulu Finland; ^3^ Circuits and Systems Research Unit, Faculty of Information Technology and Electrical Engineering University of Oulu Oulu Finland

**Keywords:** blood pressure, exercise, individual responses, near‐infrared spectroscopy

## Abstract

We hypothesized that skeletal muscle oxygenation, measured by Near‐infrared spectroscopy (mNIRS), is associated with cardiac workload during incremental exercise. Healthy subjects (*n* = 30, age 27 ± 6, 15 females) performed a maximal exercise test starting from 0 W, following an incremental protocol starting from 40 W and increasing load every 2 min until exhaustion. Systolic blood pressure and breath‐by‐breath gas exchanges were measured to analyze oxygen uptake and respiratory compensatory point (RCP). Tissue saturation index (TSI) by mNIRS was measured from vastus lateralis. The slopes of TSI and rate pressure product (RPP) were calculated using the values from 0 W to 100% of the RCP threshold. RPP was 31,734 ± 3909 mmHg·bpm, and TSI was 50.0% ± 8.4% at the intensity of RCP. RPP and TSI slopes were 3463 ± 541 and −2.75 ± 1.68, respectively. In linear regression analysis, RPP slope was used as a dependent variable, and sex, body fat %, maximal oxygen uptake, hemoglobin, baseline SBP, and TSI% slope were used as predictor variables; TSI slope was the only variable associated with RPP slope (*r* = 0.60, *p* = 0.001). Cardiac workload during submaximal exercise, documented by RPP slope calculated over equal metabolic exercise intensities for all subjects, is partly regulated by skeletal muscle oxygenation, potentially due to the differences in microcirculation and/or mitochondrial properties in healthy young subjects.

## 1 INTRODUCTION

An exaggerated blood pressure response to maximal exercise test has been shown to be related to following hypertension, cardiovascular morbidity, and mortality in follow‐up studies (Laukkanen et al., 2006; Singh et al., 1999; Weiss et al., 2010). Both maximal systolic blood pressure (SBP) and the slope of SBP response to incremental exercise test are related to subsequent cardiovascular diseases (Laukkanen et al., 2006). In healthy subjects, the SBP should increase approximately 10 mmHg per metabolic equivalent and may plateau at peak exercise (Pescatello et al., 2014; Sharman & Lagerche, 2015), while diastolic blood pressure (DBP) remains relatively constant (Manou‐Stathopoulou et al., 2015). However, large interindividual differences in SBP during exercise at the same relative exercise intensity have been observed both during static exercise and dynamic exercise in healthy subjects (Weippert et al., 2013). In addition, some people have extremely high systolic blood pressure during exercise, which is associated with cardiovascular diseases such as hypertension (Schulzand & Sharman, 2014).

Adequate blood flow is crucial for delivering oxygen to skeletal muscles and for the removal of metabolic waste products both at rest and during physical activity. This process is regulated through an increase in systolic blood pressure as well as vasodilation of microvascular vessels in skeletal muscle (Joyner & Casey, 2015). The skeletal muscle microcirculation is a network of blood vessels <150 μm in diameter, comprising arterioles, capillaries, and venules. This network is responsible for the primary function of the vascular tree and regulation of tissue perfusion for optimal exchange of gases and removal of metabolic waste products (Smits et al., 2016). Near‐Infrared Spectroscopy (mNIRS) is a widely used technique allowing the measurement non‐invasively of skeletal muscle microcirculation during exercise. The mNIRS device measures oxyhemoglobin, deoxyhemoglobin, total hemoglobin concentration changes, and tissue saturation index (TSI) in skeletal muscle tissue as measures for microcirculation and potentially a power of mitochondrial respiration (Boone et al., 2015, 2016; Dipla et al., 2017). Microcirculation may contribute to the unexplained variance in SBP during exercise (Dipla et al., 2017). In addition, sex‐related differences in microcirculatory responses during brachial artery occlusion have been observed, as measured by mNIRS (Jeskanen et al., 2024; Rasica et al., 2022). For these reasons, we wanted to investigate the relationship between cardiac workload and microcirculation changes measured by mNIRS during incremental exercise.

mNIRS parameters are changing linearly from very low exercise intensity until the submaximal exercise intensity assessed as respiratory compensation point (RCP) (Contreras‐Briceño et al., 2022). We hypothesized that skeletal muscle oxygenation, measured by mNIRS technology, may be related to SBP and rate pressure product (RPP) responses during submaximal exercise intensity. This association was examined both at a standardized relative intensity and at each absolute workload in healthy young subjects.

## 2 METHODS

### 2.1 Ethical approval

The participants received both oral and written information for the study, and signed informed consent was required for participation. The study was approved by the Ethics Committee of Oulu University Hospital District.

### 2.2 Study subjects and protocol

Healthy normotensive female (*n* = 15, 28 ± 7 years) and male (*n* = 15, 26 ± 5 years) subjects participated in this study. The inclusion criteria were as follows: BP < 130/85 mmHg for 1 week home ambulatory monitoring, body mass index <30 kg/m^2^, age 20–39, and no smoking or use of any other nicotine products.

During the first visit, anthropometrics, body fat % (InBody 720 Biospace, Seoul, Korea), and blood hemoglobin from the fingertip were measured (Hemocue Hb 201+, Hemocue AB, Sweden). Thereafter, participants performed a maximal exercise test on a bicycle ergometer (Monark 839E, Sweden) starting from 0 W (5 min), following an incremental protocol starting from 40 W and increasing the load every 2 min (15 W for females and 20 W for males) until exhaustion. The absolute workload of the ergometer was controlled by a Monark electrical device which offers a consistent load regardless of the pedaling rate. The pedaling rate started from 60 rpm and increased gradually to 90 rpm until the end of the exercise. Breath‐by‐breath gas exchanges and ventilation (Vyntus CPX; Vyaire Medical) were measured continuously to analyze oxygen uptake, ventilation, and the respiratory compensatory point (RCP). Physical strain was evaluated objectively by heart rate (HR) and subjectively by Borg's rate of perceived exertion scale from 6 to 20 (RPE). HR was monitored continuously, and perceived exertion was assessed at every load. ECG was recorded and monitored continuously using a 15‐lead ECG (Cardiosoft V6.71, GE Healthcare, Freiburg, Germany).

### 2.3 Near‐Infrared Spectroscopy (NIRS)

mNIRS provides non‐invasive techniques to measure the changes in oxygenation and hemodynamics in muscle tissue. It provides a method to measure mNIRS‐derived oxygenated hemoglobin (O_2−_Hb), deoxygenated hemoglobin (HH‐Hb), total hemoglobin (tHb) and tissue saturation index (TSI). The theory of mNIRS during exercise measurements has been described elsewhere (Ferrari et al., 2004). mNIRS measurements were performed using a continuous wave mNIRS system (Oxymon Mk III near‐infrared spectrophotometer; Artinis Medical Systems, Zetten, Netrherlands). The mNIRS probe included one receiving and two transmitting optodes operating at wavelengths of 765 and 860 nm. The probe was placed on the right leg vastus lateralis muscle (midthigh level and parallel to the long axis of the muscle) and attached to the skin by a double‐sided adhesive tape and secured by an elastic tape (Pirovano et al., 2020). The thickness of subcutaneous fat overlying the vastus lateralis muscle was measured using Harpenden skinfold calipers (CMS Instruments, London, England) and divided by two to estimate the actual adipose tissue thickness (Selkow et al., 2011). The mNIRS device was calibrated according to manufacturer specifications before each test. The interoptode distance was set to 40 mm, and the sampling frequency was set to 10 Hz. The measured mNIRS data were averaged to give values in 1‐s intervals. Baseline mNIRS values represent the mean steady‐state value of the last 60s of the unloaded 5 min cycling (0 W) before the maximal exercise test started according to Delorey et al. (DeLorey et al., 2003).

### 2.4 Analysis of mNIRS during and after exercise

**FIGURE 1:**
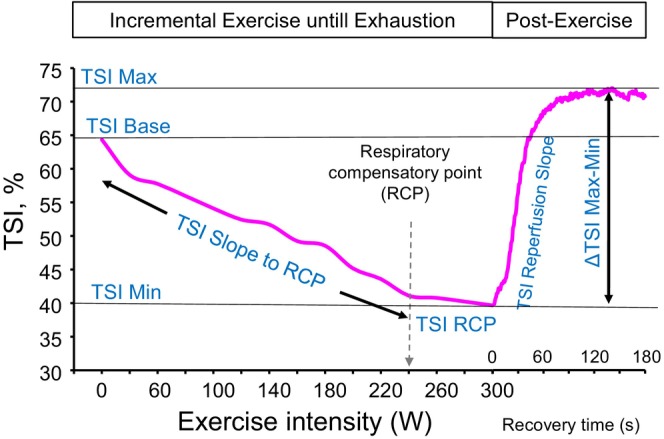
An example of tissue saturation index (TSI) dynamics during incremental exercise test and post‐exercise for one subject.

**FIGURE 2:**
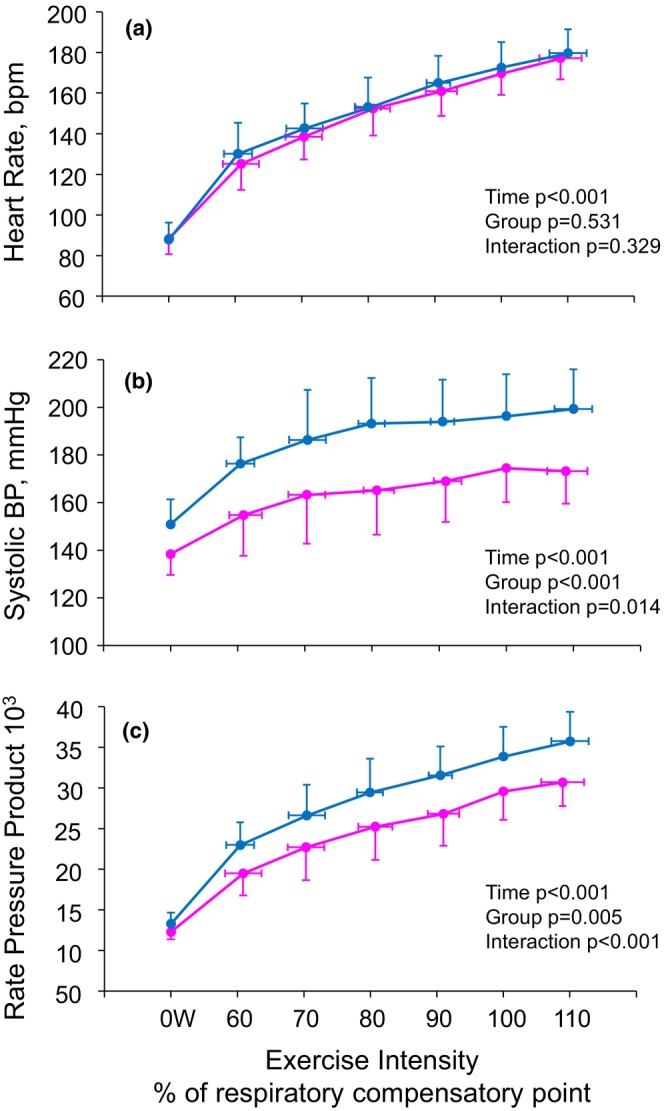
Heart rate (a), systolic blood pressure (b) and rate pressure product (c) during exercise at the equal exercise intensities calculated as a percentage of respiratory compensatory point (100%) for male (blue lines) and female (magenta lines).

**FIGURE 3:**
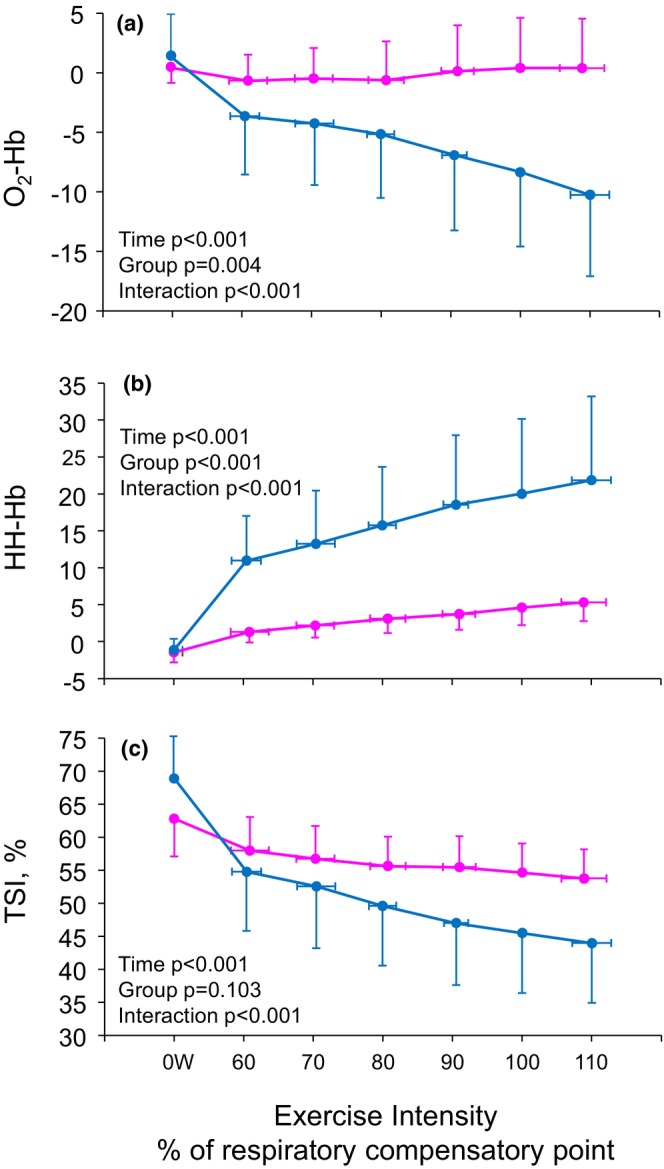
Oxyhemoglobin (a), deoxyhemoglobin (b) and tissue saturation index (c) during exercise at the equal exercise intensities calculated as a percentage of respiratory compensatory point (100%) for male (blue lines) and female (magenta lines).

TSI, oxyhemoglobin, and deoxyhemoglobin were measured continuously during the maximal exercise test. In addition, the minimum and maximum TSI values (TSI min and TSI max) were measured during and after the test. As the load increased, the subject reached the load of the respiratory compensation point (RCP), which was determined visually using minute ventilation (VE), heart rate (HR), expired carbon dioxide (VCO_2_) and oxygen (VO_2_) (Bellar et al., 2015). Especially, VE, VE/VCO_2_, and VE/VO_2_ were used to determine the RCP by comparing them with HR and VO_2_ (Bellar et al., 2015; Keir et al., 2022). The RCP analysis was verified by comparing VO_2_ at RCP to VO_2_max, which is expected to be approximately 80% (Keir et al., 2022). This relationship is presented in Table 1. Furthermore, the mNIRS values were determined for the closest different loads of RCP percentages (from 60% to 110%) to illustrate mNIRS values changes during exercise (Figures 1 and 3). This small individual range between the different RCP percentages can be seen in Figures 2 and 3. RCP‐TSI, RCP HH‐Hb, and RCP‐O2‐Hb were the values at the RCP load. Absolute changes (ΔTSI, ΔHH‐Hb and ΔO2‐Hb) were computed using the values of baseline and RCP load. The relative slopes (Slope‐TSI, Slope‐HH‐Hb and Slope‐O2‐Hb) were calculated using baseline values (0 W) and the values of 60%, 70%, 80%, 90%, and 100% of the RCP threshold. The absolute slopes (TSI, HH‐Hb and O2‐Hb) were calculated using the baseline values (0 W) and the values at each workload during exercise, up to the RCP threshold. At the point of exhaustion, the subject stopped the leg at a 90‐degree angle in a stable position, and the mNIRS parameters describing the recovery and reperfusion were measured for 3 min. Slope during the first 15 and 30 s after the test (Slope 15 and 30 s, %/s) together with ΔTSI Max‐Min, were utilized as measures of microvascular reactivity, which is defined as the capacity of blood vessels to accommodate increased blood flow (Dipla et al., 2017).

**TABLE 1:** Characteristics of the subjects at rest, during maximal exercise and at the intensity of respiratory compensatory point.

Variables	All, *n* = 30	Female, *n* = 15	Male, *n* = 15	*p*‐level
Characteristics				
Age, years	26.7 ± 6.2	27.9 ± 6.9	25.5 ± 5.3	0.294
Weight, kg	71.6 ± 11.1	64.0 ± 7.3	79.1 ± 8.9	<0.001
Height, cm	174.9 ± 8.4	168.7 ± 4.3	181.3 ± 6.8	<0.001
BMI, kg/m^2^	23.4 ± 2.2	22.6 ± 2.3	24.1 ± 1.8	0.069
Hemoglobin, g/L	137 ± 13	127 ± 9	145 ± 9	<0.001
Boby Fat, %	18.2 ± 7.1	23.2 ± 5.5	13.1 ± 4.6	<0.001
VL Thickness, mm/2	5.1 ± 1.9	5.9 ± 2.1	4.3 ± 1.4	0.025
Sys‐BP home, mmHg	111 ± 9	106 ± 9	117 ± 5	0.004
Dia‐BP home, mmHg	70 ± 6	70 ± 5	69 ± 7	0.673
Sys‐BP lab, mmHg	127 ± 13	122 ± 13	132 ± 13	0.049
Dia‐BP lab, mmHg	76 ± 6.4	76 ± 7	76 ± 6	0.985
Rest HR, beats/min	66 ± 11	70 ± 10	62 ± 11	0.029
Rest RPP lab, mmHg·bpm	8371 ± 1776	8607 ± 1743	8136 ± 1838	0.477
Exercise capacity				
VO2 peak, mL/kg/min	44.2 ± 6.9	40.7 ± 4.7	47.7 ± 7.1	0.004
Maximal load, Watts	243 ± 56	201 ± 25	285 ± 44	<0.001
Maximal RER	1.12 ± 0.04	1.13 ± 0.04	1.12 ± 0.04	0.460
Maximal HR, bpm	189 ± 10	188 ± 9	190 ± 11	0.536
RCP threshold				
Vo2% of max	80.5 ± 7.6	80.3 ± 6.9	80.7 ± 8.5	0.885
HR% of max	90.6 ± 3.6	90.5 ± 4.3	90.7 ± 3.0	0.850
HR, bpm	171 ± 11	170 ± 10	173 ± 12	0.462
RER	1.01 ± 0.05	1.00 ± 0.05	1.02 ± 0.05	0.458
RPE	15.8 ± 1.4	15.7 + 1.4	15.9 ± 1.3	0.604
Sys‐BP, mmHg	185 ± 19	174 ± 14	196 ± 17	<0.001
RPP, mmHg·bpm	31,734 ± 3909	29,585 ± 2934	33,884 ± 3626	0.001
RCP O_2_‐Hb	−4.0 ± 6.9	0.4 ± 4.2	−8.3 ± 6.2	<0.001
RCP HH‐Hb	12.3 ± 10.7	4.6 ± 2.4	20.0 ± 10.1	<0.001
RCP‐TSI	50.0 ± 8.4	54.7 ± 4.4	45.5 ± 9.1	0.002

*Note*: Values are means ± standard deviation.

Abbreviations: BMI, body mass index; BP, blood pressure; HH‐Hb, deoxyhemoglobin; HR, heart rate; O_2_‐Hb, oxyhemoglobin; Peak VO2, peak oxygen uptake; RCP, respiratory compensation point; RER, respiratory‐exchange ratio; RPE, rate of perceived exertion; RPP, rate pressure product; TSI%, tissue saturation index; VL, vastus lateralis.

### 2.5 Analysis of cardiac work during exercise

HR and SBP were measured three times before the maximal exercise test, and baseline values were determined by the average of all measurements. SBP was measured using brachial blood pressure (Schiller BP 200+, Switzerland) and rate pressure product (RPP) was calculated (HR × SPB) to describe the total cardiac work and myocardial oxygen consumption (Gobel et al., 1978). SBP was assessed at the end of each load (every 2 min) before the next load started. Additionally, HR, SBP, and RPP values were determined for different loads of RCP percentages (60%–110%) to show increased cardiac workload during exercise at the same metabolic status for each subject (Figure 2). The absolute changes (ΔHR, ΔSBP and ΔRPP) were calculated using baseline values (0 W) and the RCP values. The relative and absolute slopes (HR, SBP, RPP) were computed using the same protocol as in mNIRS slopes during exercise. Finally, young healthy men have higher systolic blood pressure compared to women in the same age (Šebeková et al., 2020). Therefore, the results were analyzed also separately for males and females.

### 2.6 Statistics

The data was analyzed using SPSS software (IBM SPSS Statistics 24; IBM Corp, Armonk, New York). The data is reported as mean ± standard deviation, and a *p*‐value of <0.05 was considered significant. The dependent variables were checked for normality (Gaussian distribution) by visual inspection and the Shapiro–Wilk test. An independent *t*‐test was used to study the difference in characteristics (Table 1) and mNIRS values (Table 2) between male and female. Analysis of variance for repeated measures with time, sex, and time × sex interaction was used to study hemodynamics (Figure 2) and mNIRS variables (Figure 3) during exercise. Pearson correlation was used to study the association between relative and absolute RPP slope and TSI slope during exercise (Tables 3 and 4). Linear regression analysis with the forward stepwise method (addition criterion: probability of F to enter ≤0.050, unstandardized β and 95% confidence interval (CI) for β are reported) was used to study the association between cardiac workload during exercise RPP slopes as a dependent variable and TSI slope, systolic blood pressure at home, age, sex, hemoglobin, body fat%, Vastus Lateralis thickness, and maximal oxygen uptake (ml/kg/min) as predictors. Regression analysis was performed separately for absolute and relative slopes.

**TABLE 2:** The absolute change and slope of hemodynamics and NIRS parameters from 0 watts to respiratory compensatory point and NIRS parameters during recovery phase of exercise (reperfusion).

Variables	All, *n* = 30	Female, *n* = 15	Male, *n* = 15	*p*‐level
Absolute change				
ΔHR, bpm	83 ± 11	81 ± 11	85 ± 11	0.403
ΔSys‐BP, mmHg	41 ± 15	36 ± 13	45 ± 16	0.083
ΔRPP, mmHg·bpm	18,933 ± 3117	17,282 ± 1902	20,584 ± 3265	0.002
ΔO2‐Hb	−4.7 ± 7.5	0.1 ± 4.3	−9.6 ± 6.8	<0.001
ΔHH‐Hb	13.6 ± 10.6	6.1 ± 3.0	21.1 ± 10.1	<0.001
ΔTSI	−15.8 ± 9.5	−8.2 ± 2.8	−23.4 ± 7.3	<0.001
Slope relative loads				
HR, bpm	15.2 ± 2.0	15.0 ± 1.9	15.4 ± 2.1	0.671
Sys‐BP, mmHg	7.1 ± 2.3	6.2 ± 1.9	8.0 ± 2.5	0.035
RPP, mmHg·bpm	3463 ± 541	3169 ± 264	3757 ± 592	0.002
O2‐Hb	−0.80 ± 1.36	0.07 ± 0.84	−1.67 ± 1.22	<0.001
HH‐Hb	2.42 ± 1.87	1.10 ± 0.54	3.74 ± 1.78	<0.001
TSI	−2.75 ± 1.68	−1.41 ± 0.46	−4.10 ± 1.33	<0.001
Slope absolute loads				
HR, bpm	9.2 ± 1.6	9.7 ± 1.4	8.7 ± 1.7	0.084
Sys‐BP, mmHg	4.6 ± 1.6	4.1 ± 1.5	5.0 ± 1.6	0.151
RPP, mmHg·bpm	2089 ± 362	2037 ± 281	2142 ± 432	0.438
O2‐Hb	−0.34 ± 0.70	0.01 ± 0.55	−0.70 ± 0.65	0.003
HH‐Hb	1.35 ± 0.96	0.71 ± 0.33	2.00 ± 0.94	<0.001
TSI	−1.58 ± 0.86	−0.97 ± 0.35	−2.20 ± 0.78	<0.001
Reperfusion				
Slope 15 s	0.64 ± 0.43	0.51 ± 0.46	0.76 ± 0.38	0.117
Slope 30 s	0.62 ± 0.32	0.45 ± 0.31	0.80 ± 0.22	0.001
TSI min, %	47.2 ± 7.8	51.8 ± 4.9	42.6 ± 7.5	<0.001
TSI max, %	70.2 ± 4.9	67.6 ± 3.4	72.9 ± 4.8	0.002
ΔTSI max‐min, %	23.1 ± 9.9	15.8 ± 4.4	30.3 ± 8.3	<0.001

*Note*: Values are means ± standard deviation.

Abbreviations: BP, blood pressure; HR, heart rate; RPP, rate pressure product; TSI%, tissue saturation index, O_2_‐Hb, oxyhemoglobin; HH‐Hb, deoxyhemoglobin.

**TABLE 3:** Pearson's correlation between cardiac workload (RPP slope), characteristics, TSI slope during relative exercise loads.

	RPP slope	Age	Sex	Fat%	VL	Vo2 peak	Hb	RER Rcp	SBP home	TSI slope
RPP Slope	1									
Age	−0.08	1								
Sex	0.55^†^	−0.20	1							
Fat %	−0.57*	0.06	−0.72^‡^	1						
VL	−0.20	−0.10	−0.41*	0.44*	1					
Vo2peak	0.54^†^	−0.05	0.52^†^	−0.72^‡^	−0.30	1				
Hb	0.41	−0.04	0.71^‡^	−0.43	−0.33	0.13	1			
RER‐rcp	0.24	0.15	0.14	0.05	0.12	−0.10	0.11	1		
SBP home	0.25	−0.27	0.58^†^	−0.37	−0.39	0.27	0.61^†^	−0.03	1	
TSI slope	−0.60^‡^	0.29	−0.81^‡^	0.70^‡^	−0.38*	−0.53^†^	−0.49*	−0.24	−0.38	1

Abbreviations: BP, blood pressure; Fat%, body fat percentage; Hb, hemoglobin; RER, respiratory‐exchange ratio; RPP, rate pressure product; TSI%, tissue saturation index; VL, vastus lateralis thickness; Vo2peak, peak oxygen uptake.

* *p* < 0.05.

^†^ *p* < 0.01.

^‡^ *p* < 0.001.

**TABLE 4:** Pearson's correlation between cardiac workload (RPP slope), characteristics, TSI slope during absolute exercise loads.

	RPP slope	Age	Sex	Fat%	VL	Vo2 peak	Hb	RER Rcp	SBP home	TSI slope
RPP Slope	1									
Age	−0.22	1								
Sex	0.15	−0.20	1							
Fat %	0.04	0.06	−0.72^‡^	1						
VL	−0.12	−0.10	−0.41*	0.44*	1					
Vo2peak	−0.31	−0.05	0.52^†^	−0.72^‡^	−0.30	1				
Hb	0.16	−0.04	0.71^‡^	−0.43	−0.33	0.13	1			
RER‐rcp	0.35	0.15	0.14	0.05	0.12	−0.10	0.11	1		
SBP home	0.21	−0.27	0.58^†^	−0.37	−0.39	0.27	0.61^†^	−0.03	1	
TSI slope	−0.48^†^	0.32	−0.73^‡^	0.57^†^	0.32	−0.35	−0.39	−0.30	−0.35	1

Abbreviations: BP, blood pressure; Fat%, body fat percentage; Hb, hemoglobin; RER, respiratory exchange ratio; RPP, rate pressure product; TSI%, tissue saturation index; VL, vastus lateralis thickness; Vo2peak, peak oxygen uptake.

* *p*< 0.05.

^†^ *p* < 0.01.

^‡^ *p* < 0.001.

## 3 RESULTS

The characteristics of participants are shown in Table 1. Males have higher peak oxygen uptake and watts during incremental exercise. Males have higher blood hemoglobin, SBP at home, and SBP at resting laboratory conditions than females, but the same cardiac workload (RPP) at resting laboratory conditions. In addition, males have a slightly smaller Vastus Lateralis Thickness (4.3 ± 1.4 vs. 5.9 ± 2.1, *p* = 0.025). Males have higher SBP (174 ± 14 vs. 196 ± 17 mmHg, *p* < 0.001) and RPP (29,585 ± 2934 vs. 33,884 ± 3626 mmHg·bpm, *p* = 0.001) at the same exercise intensity defined as RCP. However, there were no differences in workload at the intensity of RCP measured objectively by relative oxygen uptake, absolute and relative HR, RER, or workload measured subjectively by RPE (Table 1). Males also have a marked lower TSI and O2‐Hb and higher HH‐Hb levels than females at the intensity of RCP (Table 1).

The absolute change and relative/absolute slopes of hemodynamics and mNIRS parameters from 0 watts to the intensity of RCP are shown in Table 2. There were no significant differences between male and female in HR analyzed as absolute change (81 ± 11 vs. 85 ± 11 bpm, *p* = 0.403), relative slope (15.0 ± 1.9 vs. 15.4 ± 2.1, *p* = 0.671) or absolute slope (9.7 ± 1.4 vs. 8.7 ± 1.7, *p* = 0.084). However, the change in cardiac workload (RPP) was higher in male than female analyzed in absolute units (17,282 ± 1902 vs. 20,584 ± 3265 mmHg·bpm, *p* = 0.002) or relative slope techniques (3169 ± 264 vs. 3757 ± 592, *p* = 0.002). In addition, the relative SBP slope was higher in male than female (6.2 ± 1.9 vs. 8.0 ± 2.5, *p* = 0.035). Similarly, the TSI decreased significantly more in males compared to females analyzed in absolute units (−23.4 ± 7.3 vs. −8.2 ± 2.8, *p* < 0.001), relative slope techniques (−4.10 ± 1.33 vs. −1.41 ± 0.46, *p* < 0.001) and absolute slope technique (−2.20 ± 0.78 vs. −0.97 ± 0.35, *p* < 0.001). All reperfusion parameters except Slope 15 s after exercise differ between male and female, e.g. reperfusion slope 30 s was 0.80% ± 0.22%/s vs. 0.45% ± 0.31%/s (*p* < 0.001) for male and female, respectively.

The changes in hemodynamics and mNIRS parameters for male and female at baseline (0 W) and from 60% to 110% of RCP are shown in Figures 2 and 3. There were no differences in HR (time × sex interaction *p* = 0.329), but SBP (time × sex interaction *p* = 0.014) and cardiac workload (time × sex interaction *p* < 0.001) were lower in females than males at the same relative intensities (Figure 2). O_2_‐Hb and TSI were lower and HH‐Hb higher in males compared to females (Figure 3).

Pearsons´ correlation between cardiac workload during exercise both relative and absolute scales (RPP slopes), participants´ characteristics, and relative and absolute TSI slope during exercise are shown in Tables 3 and 4. Relative RPP Slope was correlated with sex, fat %, peak oxygen uptake and relative TSI Slope during exercise (Table 3). Absolute RPP slope was correlated only with absolute TSI slope (Table 4).

**FIGURE 4:**
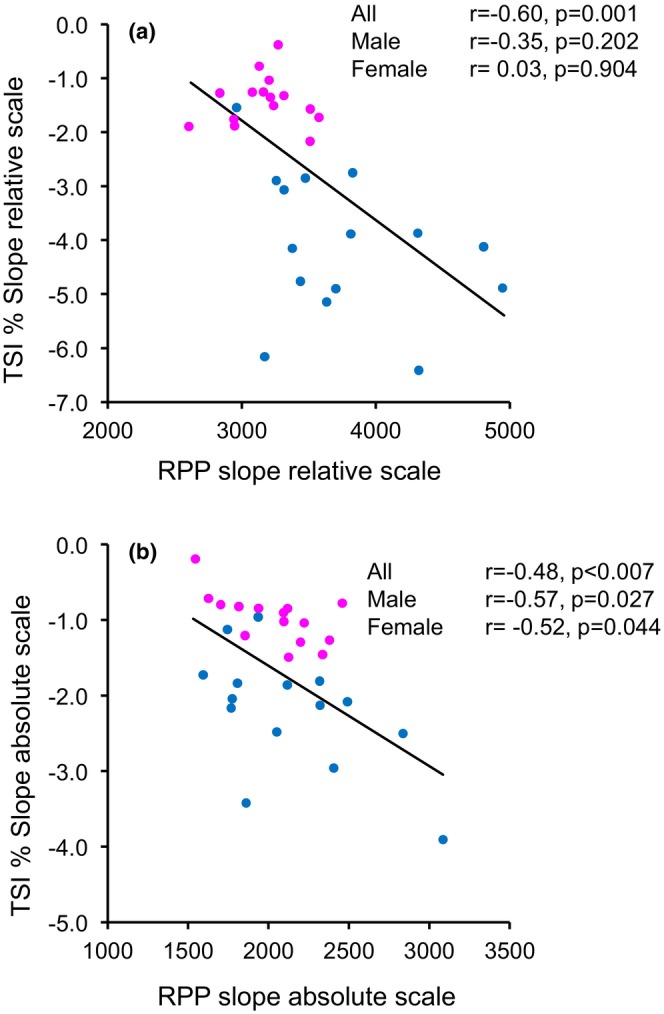
The correlation between the relative slope of rate pressure product (RPP) and the relative slope of tissue saturation index (TSI) calculated from 0 W to 100% of respiratory compensatory point (RCP) (a). Absolute slope of RPP and TSI calculated using 0 W values and each absolute load values to RCP (b). Blue dots for male and magenta dots for female.

In linear regression analysis, relative RPP slope as a dependent variable and sex, body fat%, Vastus Lateralis thickness, peak oxygen uptake, hemoglobin, SPB at home, and relative TSI slope, relative TSI slope was the only variable associated with relative RPP slope (*R* = 0.60, adjusted *R* ^2^ = 0.33, *p* = 0.0005). In the same linear regression analysis for absolute RPP slope, the absolute TSI slope (partial correlation *r* = −0.67, β = −282, 95% CI −408 to −157, *p* = 0.0008) and peak oxygen uptake (partial correlation *r* = −0.58, β = −28, 95% CI −44 to −13, *p* = 0.0008) remained in the model (*R* = 0.70, adjusted *R* ^2^ = 0.46, *p* = 0.0005). The Pearson correlation between RPP relative slope and TSI relative slope is shown in Figure 4a and for absolute slopes in Figure 4b, for all participants and separately for female and male.

## 4 DISCUSSION

The main finding of this study is that cardiac workload during submaximal exercise analyzed in relative scales was moderately but significantly associated with the skeletal muscle tissue saturation index during exercise independently of any other variables including sex and exercise capacity. Analyzed in absolute scales, cardiac workload was associated with the tissue saturation index and exercise capacity. Elevated cardiac workload during exercise may be triggered by altered skeletal muscle oxygenation potentially due to the differences in microcirculation and/or mitochondrial function even in healthy young subjects. Secondly, cardiac workload is markedly higher in males compared to females at the equal submaximal exercise intensity, documented objectively by relative oxygen uptake and respiratory‐exchange ratio, and subjectively by Borg's rate of perceived exertion scale.

### 4.1 Cardiac workload during exercise

During the exercise, sympathetic activity starts to dominate over vagal tone to increase HR, cardiac contractility and SBP resulting in increased cardiac output but reduces systemic vascular resistance (Manou‐Stathopoulou et al., 2015). Blood flow is directed from low‐priority visceral areas to the skeletal muscles. SBP is increasing progressively as workload increases and having highest peak at maximal workload (Le et al., 2008; Myers, 1994). Additionally, the peak and the change of SBP is higher in men compared to women (Wielemborek‐Musial et al., 2016).

HR increases linearly to deliver and utilize oxygen in working muscle tissues during exercise (Myers, 2001). The maximal HR declines with age, but gender does not affect maximal HR during exercise (Nes et al., 2013). RPP increased also during exercise due to the increase in HR and SBP. These changes in HR, SBP, and RPP can be seen in Figure 2. Well‐trained young males can have a SBP up to 230 mmHg during maximal exercise intensity (Heimark et al., 2022) and well‐trained male athletes have also higher SBP compared to women athletes during exercise (Pressler et al., 2018). In addition, untrained young individuals have higher SBP during exercise compared to well‐trained young individuals (Nakamura et al., 2021). Thus, males greater SBP and RPP cannot be explained by physical condition. Higher SBP in men may be explained by slight differences in the SBP regulation system between men and women. Males have different sympathetic nervous system activation compared to females (Joyner et al., 2016) and the role of different hormones such as testosterone and estrogen can explain men's higher SBP (Reckelhoff, 2001). In the present study, RER, RPE, Vo2% of max, HR% of max, and HR were not significant at the RCP threshold (Table 1). Therefore, it can be thought that the subjects were at the same metabolic level at the RCP point and that cannot explain the absolute change in SBP and RPP between genders (Table 2, Figure 2). When comparing relative and absolute changes in cardiac workload, no statistically significant sex differences were observed. However, a slight trend was noted, suggesting that women may need to increase heart rate more than men at absolute workloads (Table 1). This tendency could be attributed to the higher VO2peak typically observed in men or potentially to lower cardiac performance in women, which may be compensated by an elevated heart rate. Similar findings have been reported in a previous study (Wheatley et al., 2014). Moreover, their findings demonstrate similar trends and sex‐related differences in SBP responses during exercise, consistent with our results (Wheatley et al., 2014).

### 4.2 Muscle oxygenation during exercise

Most of the recent studies have examined the mNIRS technique to investigate skeletal muscle oxygenation and microvascular function during arterial occlusion. Previous research has demonstrated that males have a greater change and that exercise training improves muscle oxygenation during occlusion (Jeskanen et al., 2024; Rasica et al., 2022). However, mNIRS has been proved to have high reproducibility also during exercise (Austin et al., 2005). It should be noted that men's greater muscle oxygenation is independent of the mNIRS device location because (Dellinger et al., 2023) showed the same measured muscle oxygenation in the leg.

In our knowledge, there are not many studies that have investigated changes in mNIRS parameters and SBP during incremental exercise protocol. It has been proved that TSI decreases progressively as walking speed increases, regardless of gender and age (Landers‐Ramos et al., 2024). They also found that TSI is lower when the walking speed is related to age in older compared to young individuals. This may be because the capacity for muscle oxygenation and microvascular function is reduced with age (Tonson et al., 2017).

We found that all mNIRS parameters change linearly during exercise and males had greater changes compared to females (Figure 3). The same has been observed also during an incremental cycle exercise by Espinosa‐Ramírez et al., (2021). In addition, the changes are the same in different skeletal muscles (Chin et al., 2011). The present study proves firstly that TSI, O_2_Hb, and HHb change during exercise. Secondly, there is also a significant difference between both relative and absolute mNIRS slopes during exercise between males and females (Table 2). Therefore, it may be inferred that mNIRS changes during exercise are independent of whether the workload is absolute or relative to individual metabolic thresholds. Additionally, the slope method has been proved to be the best method to assess the intramuscular oxygenation level as a function of workload (Agbangla et al., 2017). The reason behind this may be males bigger muscle mass, greater muscle oxygenation capacity, or the greater peripheral workload and gender differences between anatomical and function in the primary muscle groups recruited during maximal exercise (Espinosa‐Ramírez et al., 2021).

After maximal exercise test, TSI rapidly increases, which can be seen in Figure 1 (post‐exercise phase). Recent studies have observed differences in microvascular reactivity between males and females after arterial occlusion (Dellinger et al., 2023; Rasica et al., 2022) and between trained and untrained subjects (Brizendine et al., 2013; Rasica et al., 2022). More studies are needed to assess mNIRS parameters after exercise, but it seems that gender and training may be in the key role to explain the differences, including mitochondrial capacity and microvascular function.

### 4.3 Association between cardiac workload and muscle oxygenation during exercise

We found that muscle TSI negatively correlates with SBP and RPP, suggesting that greater peripheral oxygen extraction or variation in local skeletal muscle oxygenation dynamics may be accompanied by a higher cardiac workload. These differences may be influenced by different types of muscle fibers (fast or slow muscle fibers) and/or individual variability in mitochondrial function. There are no recent studies to examine or explain the connection between muscle TSI and cardiac workload. In addition, linear regression analysis showed that relative TSI slope was the only variable associated with relative RPP slope, proving that sex, physical condition, hemoglobin, or SBP at home cannot explain the association. Absolute RPP slope was associated with both the absolute TSI slope and peak oxygen uptake (VO_2_peak). This finding is physiologically consistent, as individuals with higher Vo2peak can sustain exercise for a longer duration and typically reach higher absolute workloads before reaching the RCP threshold. Consequently, greater workloads may require a proportionally higher cardiac workload. This connection between relative/absolute RPP slope and TSI slope may be explained also by individual autonomic nervous system function response to exercise (Dela et al., 2003; Manou‐Stathopoulou et al., 2015) causing differences in cardiac output and peripheral vasodilation.

Further future studies are needed to understand the association between cardiac workload and muscle oxygenation better. On the other hand, mNIRS can help to develop training and testing in sport medicine. We can determine RCP (Contreras‐Briceño et al., 2022) and lactate threshold (Snyder & Parmenter, 2009) by using NIRS. In addition, mNIRS has proved to assess better the training intensity compared to heart rate (Born et al., 2017) and be a useful method to monitor aerobic training (Klusiewicz et al., 2021). Dunst et al., (2023) and Porter & Langley (2025) demonstrated that mNIRS reliably predicts blood lactate concentrations during short, high‐intensity exercise. Therefore, mNIRS appears to be a valuable technology for monitoring anaerobic exercise. Future researchers should utilize mNIRS to advance knowledge of skeletal muscle oxygenation and function during both aerobic and anaerobic exercise.

Our study suggests that at the same metabolic level, males' total cardiac load is significantly larger due to greater SBP and RPP, and the TSI is lower in skeletal muscle tissue compared to women. These observations may have implications for individualized training approaches in the future. Finally, the steeper RPP slope was associated specifically with TSI slope, both relative and absolute loads, which may reflect individual physiological response to exercise.

## AUTHOR CONTRIBUTIONS

This study was conducted in 2022–2024 in the University of Oulu, Oulu, Finland. Conception and design of the work: Tulppo P. Mikko, Valtonen I.P Rasmus, Ylinen P. Venla, and Nissinen Jan. Acquisition and analysis and interpretation of the data for the work: Jeskanen Tommi, Tulppo P. Mikko, Valtonen I.P Rasmus, Ylinen P. Venla, and Nissinen Jan. Drafting the work and revising it critically for important intellectual content: Jeskanen Tommi, Tulppo P. Mikko, Valtonen I.P Rasmus, Ylinen P. Venla, and Nissinen Jan. Final approval of the version to be published: Jeskanen Tommi, Tulppo P. Mikko, Valtonen I.P Rasmus, Ylinen P. Venla, and Nissinen Jan.

Agreement to be accountable for all aspects of the work: Jeskanen Tommi, Tulppo P. Mikko, Valtonen I.P. Rasmus, Ylinen P. Venla, and Nissinen Jan.

## CONFLICT OF INTEREST STATEMENT

The authors declare no conflicts of interest.

## Data Availability

The data that support the findings of this article are not publicly available due to privacy and ethical concerns. They can be requested from the author at mikko.tulppo@oulu.fi.
